# Measurement of the Loss and Depolarization Probability of UCN on Beryllium and Diamond Like Carbon Films

**DOI:** 10.6028/jres.110.039

**Published:** 2005-06-01

**Authors:** Tomas Brys, Manfred Daum, Peter Fierlinger, Peter Geltenbort, Mukul Gupta, Reinhold Henneck, Stefan Heule, Klaus Kirch, Mikhail Lasakov, Russel Mammei, Mark Makela, Axel Pichlmaier, Anatoli Serebrov, Ulrich Straumann, Robert B. Vogelaar, Cedric Wermelinger, Albert Young

**Affiliations:** Paul Scherrer Institut, 5232 Villingen PSI, Switzerland and ETH Zürich, Zurich, Switzerland; Paul Scherrer Institut, 5232 Villingen PSI, Switzerland; Paul Scherrer Institut, 5232 Villingen PSI, Switzerland and Universitaet Zürich, Zurich, Switzerland; Institut Laue Lagevin, Grenoble, France; Paul Scherrer Institut, 5232 Villingen PSI, Switzerland; Paul Scherrer Institut, 5232 Villingen PSI, Switzerland; Paul Scherrer Institut, 5232 Villingen PSI, Switzerland and Universitaet Zürich, Zurich, Switzerland; Paul Scherrer Institut, 5232 Villingen PSI, Switzerland; PNPI Gatchina, Russia; Virginia Tech, Blacksburg, USA; Virginia Tech, Blacksburg, USA; Paul Scherrer Institut, 5232 Villingen PSI, Switzerland; Paul Scherrer Institut, 5232 Villingen PSI, Switzerland and PNPI Gatchina, Russia; Universitaet Zürich, Zurich, Switzerland; Virginia Tech, Blacksburg, USA; ETH Zürich, Zurich, Switzerland and Paul Scherrer Institut, 5232 Villingen PSI, Switzerland; North Carolina State University, Raleigh, USA

**Keywords:** depolarization, diamond like carbon, spin flip, ultracold neutrons

## Abstract

Currently several institutes worldwide are working on the development of a new generation of ultracold neutron (UCN) sources. In parallel with source development, new materials for guiding and storage of UCN are developed. Currently the best results have been achieved using ^58^Ni, Be, solid O_2_ and low temperature Fomblin oil (LTF). All of these materials have their shortcomings like cost, toxicity or difficulty of use. A novel very promising material is diamond like carbon (DLC). Several techniques exist to coat surfaces, and industrial applications (e.g., for extremely hard surfaces) are already wide spread. Preliminary investigations using neutron reflectometry at PSI and Los Alamos yielded a critical velocity for DLC of about 7 m/s thus comparable to Beryllium. A low upper limit of depolarization probability for stored polarized UCN has been measured at the PF2 facility of the Institut Laue-Langevin (ILL) by North Carolina State University (NCSU), Los Alamos National Laboratory (LANL), and Petersburg Nuclear Physics Institute (PNPI), thus making it also a good material for storage and guidance of polarized UCN. Still missing is the loss probability per bounce. We will be able to extract this number and a more stringent value for the depolarization from our experiment thus proving the suitability of DLC as a wall material for a wide range of UCN applications.

## 1. Introduction

Currently, strong efforts are under way worldwide to build new sources for ultracold neutrons (UCN)(see e.g., [1], [2], [3]). All of these new sources will use solid deuterium (sD_2_) as neutron moderator. Hand-in-hand with the development of these sources, new materials to store and guide the UCN are also required. The material of choice is Beryllium since it has a high critical velocity of 6.9 m/s, equal to Nickel and second to isotopically enriched ^58^Ni (*v*_crit_ = 8.1 m/s). Nickel however is magnetic and is not suitable for polarized UCN. Beryllium on the other hand is toxic and therefore difficult to machine and handle. One promising alternative material is diamond-like carbon (DLC) which is non-toxic, hydrophobic, stable, and widely used in industrial applications. Existing technology can be used allowing a cost effective production. Experience with DLC already exists [4], [5]. At SINQ we have recently measured the critical velocity of DLC and found it to be (7.0 × 0.3) m/s, similar to Beryllium. The quality of the coatings was further investigated by thermal cycling between 450 K and 77 K and gave stable results; the adhesion to the substrate turned out to be excellent. Contamination with other elements, especially Boron, is a concern but depends directly on the quality of the raw material used. It is small and well under control.

Scientifically most interesting is a measurement of the depolarization probability per bounce. It has been investigated in earlier experiments for DLC [5] and for Be [6] during storage. It was found to be below 3 × 10^−6^ per bounce for DLC and around 2 × 10^−5^ to 3 × 10^−5^ for Beryllium. A more detailed investigation of the spin flip probability as a function of energy and temperature, which we propose here, is important in order to

- clarify whether there is indeed a connection between spin flip and “anomalous losses,” as has been proposed recently ([6], [7]). In this picture, some UCN are spin flipped upon reflection due to incoherent scattering from para/ferro-magnetic centers on the material surface. The fraction of UCN scattered incoherently into the material could possibly explain the observed “anomalous losses.” In order to study this experimentally, one has to measure both parameters, the depolarization and the loss probability per wall collision, simultaneously in the same experiment;
- to supply information on the polarization stability during storage. Precision UCN experiments rely on the fact that the change of polarization during storage does not introduce systematic errors. Obviously, improving the accuracy for the planned experiments also places stronger constraints on the control of the polarization. Although the sensitivity to depolarization effects can be inherently low, depolarization aspects need to be studied for future high-sensitivity studies [2].

Contrary to the other depolarization experiments our set-up has no mechanical slits. Although other experiments typically found wall losses in the order of 5 × 10^−5^ per bounce, theory predicts values two orders of magnitude lower [8]. The experiment thus allows to pin down both, the loss and the depolarization probability per bounce.

We use a vertical storage tube sealed on the top by gravity and on the bottom by a conventional 1.5 T magnet, corresponding to a neutron energy of 90 neV. The neutrons have no contact to any material surface other than the coating (DLC or Be). Since earlier experiments have been crippled by poor vacuum conditions, special care is taken to achieve the best possible vacuum. We use well established vacuum techniques like baking and usage of a cryo cooler for hydrogen pumping. We are able to adjust the temperature between 40 K and 450 K.

## 2. Experimental Set up and Expected Results

**Fig. 1:**
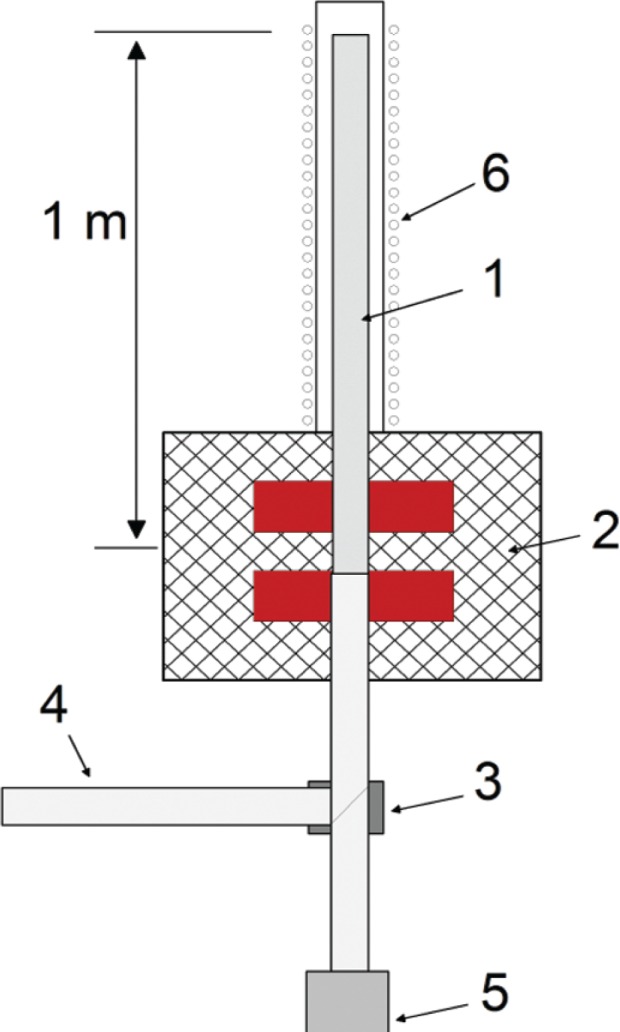
Set up of the experiment. 1 — sample tube, 2 — magnet, 3 — beam switch, 4 — feeding guide, 5 — UCN detector, 6 — holding field coil.

**Fig. 2:**
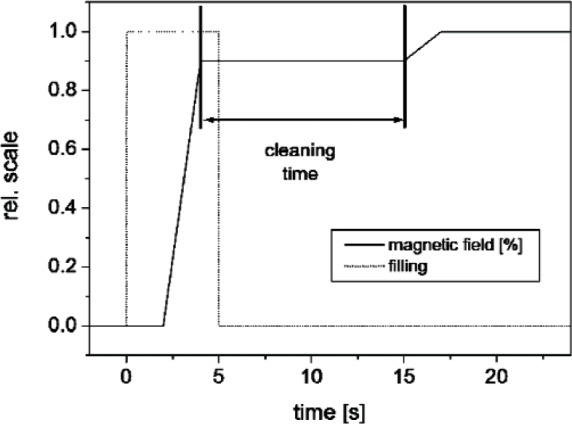
Ramping of the magnetic field during filling and spectral cleaning. The field is ramped up to 90 % of the maximum value while the neutrons are filled into the storage volume. Neutrons with “wrong” spin state or too high energy are effectively removed during the cleaning time. The magnet is then ramped to maximum field and the storage measurement starts.

The experiment is carried out at the ILL-PF2 ultra cold neutron installation. It works as follows: UCN from the turbine enter the storage volume (1, see Fig. 1) through the feeding guide (4), an Aluminum window and a beam switch (3). At this point the magnet (2) is still switched off, but the 10^−2^ T (100 G) holding field (6) in the storage volume is already on. After equilibrium neutron density is reached, the magnet is turned on. This can be done in about 3 s. Now the beam switch is moved such that the storage volume is connected to the detector (5). Neutrons with the “wrong” spin state drain quickly from the storage volume while the others are confined by the magnetic field, the coated walls and gravity (see Fig. 2). After a certain holding time the magnet is switched off and the stored neutrons fall into the detector. This procedure is repeated with two different holding times (*t*_1_, *t*_2_) with the number of stored neutrons *N_t_*_1_ and *N_t_*_2_, respectively, thus yielding the neutron lifetime in the storage volume *τ*_tot_ Eq. (1):

$$\tau_{\text{tot}}=\frac{t_{1}-t_{2}}{\ln\left(N_{t1}/N_{t2}\right)}.$$ (1)

Using *τ*_tot_ and the total number of wall collisions ν determined by Monte Carlo simulations, the loss probability µ per wall interaction can be calculated Eq. (2).

$$\frac{1}{\tau_{\text{tot}}}=\frac{1}{\tau_{n}}+\upsilon \beta +\upsilon \mu .$$ (2)

Neutrons that experience a spin flip (N_sp_) during the storage are no longer confined by the magnetic field. They are counted in the detector during the holding time. The spinflip probability *β* can be calculated according to Eq. (3).

$$N_{\text{sp}}=(N_{t1}-N_{t2})\cdot \tau_{\text{tot}}\cdot \upsilon \cdot \beta .$$ (3)

Since the background count rate is in the order of only a few ms^−1^, and since the “wrong” spin state drains within seconds from the storage volume, we detect a clear signal from the depolarized neutrons.

Alternatively we fill the storage volume with the magnet on and store only depolarized UCN. This serves as a complementary measurement cycle which helps to exclude systematic uncertainties. It must give the same result if all parameters are well under control.

With a loss factor *µ* = 10^−4^ and a bottle lifetime of 180 s, filling and emptying time of around 2 s can be expected. This enables us to get a ≅15 % statistical accuracy for the storage time *τ* and ≅20 % for the spin flip probability *β* at *β* = 3 × 10^−6^ in one measurement cycle of about 15 min.

We will test DLC coatings made by two different techniques on Quartz and Aluminum substrates. For reference we will also measure Beryllium coated surfaces.
